# Heterologous production of the adhesin LIC13411 from pathogenic *Leptospira* facilitates binding of non-pathogenic *Leptospira in vitro* and *in vivo*

**DOI:** 10.3389/fcimb.2022.917963

**Published:** 2022-07-22

**Authors:** Matthew C. Surdel, Beth L. Hahn, Phillip N. Anderson, Jenifer Coburn

**Affiliations:** ^1^ Department of Medicine, Division of Infectious Diseases, Medical College of Wisconsin, Milwaukee, WI, United States; ^2^ Department of Microbiology and Immunology, Medical College of Wisconsin, Milwaukee, WI, United States

**Keywords:** *Leptospira*, infectious disease, bacteria, adhesin, VE-cadherin, endothelial cells, murine model

## Abstract

Leptospirosis is an important cause of morbidity and mortality worldwide. Disease severity ranges from asymptomatic colonization to widespread hemorrhage and multiorgan dysfunction. The causative agents, *Leptospira* spp., are zoonotic Gram-negative spirochetes. One important step in pathogenesis is binding of bacterial adhesins to host components. Previously our laboratory identified two *L. interrogans* candidate adhesins, LIC11574 and LIC13411, that bind to VE-cadherin *in vitro*. In the current study, we demonstrate the ability of two strains of pathogenic *L. interrogans* to disrupt the localization of VE-cadherin, a protein important to maintaining inter-endothelial junctions. Purified MBP-LIC11574 and MBP-LIC13411 bind human dermal microvascular endothelial cells in a pattern reminiscent of VE-cadherin, but do not disrupt VE-cadherin localization. Genes encoding the candidate adhesins from pathogenic *Leptospira* were cloned in an overexpression vector and introduced into non-pathogenic *L. biflexa*, creating gain-of-function strains producing LIC11574 or LIC13411. Protein production and localization to the outer membrane were confirmed by Triton X-114 fractionation. Although these strains do not disrupt VE-cadherin localization, production of LIC13411 increases binding of non-pathogenic *Leptospira* to human endothelial cells and specifically to VE-cadherin. In a short-term murine model of infection, LIC13411 production led to increased burdens of the non-pathogen in the lung, liver, kidney, and bladder. These data confirm the role of LIC13411 as an adhesin in *Leptospira* spp. and implicate it in dissemination to multiple organs. Importantly, anti-adhesin therapy has been shown to have many benefits over classical antibiotics. Taken together, this work provides novel insight into the pathogenesis of *Leptospira* spp. and identifies LIC13411 as a potential prophylactic and therapeutic target.

## Introduction

Leptospirosis is a global and potentially fatal zoonotic disease. Disease prevalence is highest in resource-poor areas; however, leptospirosis affects humans and animals in nearly all geographic and economic regions (Costa et al., 2015; reviewed in Levett, 2001; Bharti et al., 2003; Ko et al., 2009; Adler and de la Pena Moctezuma, 2010; Evangelista and Coburn, 2010; Hartskeerl et al., 2011; Picardeau, 2013; Adler et al., 2014). Recent estimates suggest that there are over one million cases of leptospirosis annually worldwide, resulting in approximately 60,000 deaths. This number is likely to be underestimated due to misdiagnosis and lack of surveillance in many regions (Abela-Ridder et al., 2010; Costa et al., 2015). Clinical disease was first described by Arthur Weil in 1886 as a form of infective jaundice (Weil, 1886; Alston and Brown, 1937). Since then, a more complete description of leptospirosis has emerged. Leptospirosis severity ranges from a mild non-specific febrile illness to a fulminant life-threatening disease characterized by multiorgan dysfunction and hemorrhage (Costa et al., 2015; Hochedez et al., 2015; Torgerson et al., 2015; reviewed in Adler and de la Pena Moctezuma, 2010; Picardeau, 2013; Haake and Levett, 2015; Picardeau, 2017). In addition to direct effects on human health, leptospirosis also affects animals, including dogs, horses, and cattle, leading to numerous indirect costs to human well-being (Bolin, 1996; Rohrbach et al., 2005; reviewed in Ellis, 1994; Adler and de la Pena Moctezuma, 2010; Adler et al., 2014).

Leptospirosis is caused by organisms of the genus *Leptospira*. *Leptospira* spp. are Gram-negative spirochetes that are characterized by their high motility, small size of less than 0.1 µm in diameter, and unique hook-shaped ends. Classically, the genus *Leptospira* was categorized into disease-inducing pathogens and non-pathogenic saprophytes. With the advent of genomic sequencing and new genetic tools to study bacterial pathogens, it is now clear that there are at least 64 species classified into three lineages: non-pathogenic saprophytes, intermediates, and pathogens (Perolat et al., 1998; Vincent et al., 2019). Many genetic differences between species in these groups have led to further characterization of factors important for pathogenesis.

Pathogenic leptospires are found in a wide range of hosts where they colonize the proximal tubules of the kidneys and are intermittently excreted in urine (Jobbins and Alexander, 2015; Mgode et al., 2015; reviewed in Adler et al., 2014). Once excreted, *Leptospira* spp. can survive for months in water and moist soil (Trueba et al., 2004; Andre-Fontaine et al., 2015; Bierque et al., 2020). Humans are accidental hosts and become infected with pathogenic *Leptospira* upon exposure to infected animals or contaminated environmental sources. Leptospires enter the host through abrasions in the skin or contact with mucous membranes (reviewed in Ko et al., 2009; Adler et al., 2014; Haake and Levett, 2015). Despite the global burden of disease, the pathogenic mechanisms of *Leptospira* remain poorly understood.

Most bacterial pathogens must bind to the host to induce disease. Gram-positive and Gram-negative bacteria express a wide variety of adhesins involved in bacterial binding to host molecules and cells (reviewed in Marra and Isberg, 1996; Niemann et al., 2004; Pizarro-Cerda and Cossart, 2006; Patel et al., 2017). The combination of adhesins expressed by a bacterium determines its tropism. Adhesins can serve multiple roles, but their basic function is to bind cells and other host components. Upon binding, the spatial localization can allow virulence determinants, such as secretion systems, to target cells and deliver effector molecules. In addition, adhesins can trigger responses within the bacteria and the host. For example, the proteins invasin and YadA from *Yersinia* spp. are involved in binding as well as uptake into mammalian cells (Isberg et al., 1987; Eitel and Dersch, 2002). The *Fusobacterium nucleatum* adhesin FadA binds to host cells through interaction with vascular endothelial (VE-) cadherin and induces the re-localization of VE-cadherin, ultimately increasing the permeability of endothelial layers (Fardini et al., 2011).

Due to the importance of adhesion in bacterial pathogenesis, therapeutic strategies have been developed to inhibit the ability of pathogens to bind host molecules. These approaches target virtually every step of the adhesion processes, from synthesis of bacterial adhesins to alteration of host receptors (Okuda et al., 2010; Huebinger et al., 2016; reviewed in Svensson et al., 2006; Krachler and Orth, 2013; Stones and Krachler, 2016; Asadi et al., 2019). One promising strategy is the targeting of bacterial adhesins with antibodies, through either passive or active immunization (Sheth et al., 1995; Huebinger et al., 2016). Since targeting adhesion does not affect bacterial viability, very low levels of resistance have been identified (reviewed in Krachler and Orth, 2013). Several potential adhesins have been identified in *Leptospira*; however, the specific roles of these proteins during infection remain to be elucidated (Evangelista et al., 2014a; Evangelista et al., 2014b; Robbins et al., 2015; reviewed in Adler, 2014; Fernandes et al., 2016).

To disseminate within the host, *Leptospira* spp. gain access to the blood stream, where they travel to target organs. In severe leptospirosis, disrupting endothelial cell barriers leads to massive hemorrhage which can result in death (Nicodemo et al., 1997; Nally et al., 2004; Hochedez et al., 2015; reviewed in Bharti et al., 2003; Adler and de la Pena Moctezuma, 2010; Haake and Levett, 2015). Proper endothelial function is therefore critical to host defense against pathogenic *Leptospira*. Adherens junctions (AJs) are a crucial component to maintaining tight endothelial barriers and are made up of numerous proteins including VE-cadherin (Carmeliet et al., 1999; reviewed in Dejana, 1996; Dejana et al., 2008; Harris and Tepass, 2010; Harris and Nelson, 2010). The importance of VE-cadherin in regulating vascular permeability and leukocyte transmigration has been widely studied. VE-cadherin production, phosphorylation, and endocytosis are a few of the ways the dynamic barrier function is maintained (Baumeister et al., 2005; Potter et al., 2005; Gavard and Gutkind, 2006; Allingham et al., 2007; Wallez et al., 2007; Nottebaum et al., 2008; Turowski et al., 2008; Monaghan-Benson and Burridge, 2009; Adam et al., 2010; Orsenigo et al., 2012; Timmerman et al., 2012; Sidibe and Imhof, 2014; Wessel et al., 2014; Garrett et al., 2017; reviewed in Dejana et al., 2008; Harris and Nelson, 2010). Furthermore, upon endocytosis VE-cadherin is degraded and VE-cadherin fragments can be identified (Su and Kowalczyk, 2017). During infection, it is likely that *Leptospira* must produce adhesins that bind to endothelial cells. These interactions likely bring the bacteria in proximity with junctional complexes that are ultimately disrupted in order to facilitate dissemination, promote colonization, and induce disease. Within the kidney, these interactions are essential to the bacterial lifestyle.

Previously, our laboratory has shown that pathogenic *L. interrogans* binds more efficiently to endothelial cells than does non-pathogenic *L. biflexa* (Evangelista et al., 2014a). Pathogenic *Leptospira* spp. induce qualitative changes in VE-cadherin staining *in vitro* as seen by immunofluorescence (IF) microscopy (Sato and Coburn, 2017). Two proteins from *Leptospira interrogans* sv. Copenhageni, LIC11574 and LIC13411, specifically bind to VE-cadherin with single-digit nanomolar affinities (Evangelista et al., 2014b). In the current study, we demonstrate that disruption of VE-cadherin localized to AJs is a conserved phenotype in two pathogenic *Leptospira* strains, suggesting that it is an important step in pathogenesis. Proteins LIC11574 and LIC13411 were heterologously produced in *L. biflexa* sv. Patoc. Although the adhesin-producing strains do not disrupt VE-cadherin localization in AJs, the strain producing LIC13411 increases binding of non-pathogenic *Leptospira* to human endothelial cells and specifically to VE-cadherin. Utilizing a recently developed short-term model of infection, LIC13411 production in the non-pathogen increases the association of the non-pathogen with multiple organs, implicating it in the pathogenesis of *L. interrogans*. Our data indicate that a VE-cadherin-binding adhesin, LIC13411, of *L. interrogans* is sufficient to increase binding of non-pathogenic *Leptospira* and to increase bacterial burdens in a mouse model of infection.

## Materials and methods

### *Leptospira* strains and growth conditions

*L. interrogans* sv. Copenhageni and *L. biflexa* sv. Patoc were gifts from David Haake (UCLA, Los Angeles, CA). *L. interrogans* sv. Manilae was a gift from Elsio Wunder (Yale, New Haven, CO). All leptospiral strains were grown at 30°C in Ellinghausen–McCullough–Johnson–Harris (EMJH) basal medium (BD Difco, Cat. No. 279410) supplemented with 0.25% glycerol, 1% rabbit serum, 0.1% lactalbumin hydrolysate, 0.0001% superoxide dismutase, 0.004% sodium pyruvate, 1% bovine serum albumin, 0.001% calcium chloride, 0.001% magnesium chloride, 0.0004% zinc sulfate, 0.00003% copper sulfate, 0.0005% ferrous sulfate, 0.00002% vitamin B12, 0.0005% thiamine chloride, and 0.125% Tween-80 (Ellinghausen and McCullough, 1965; Johnson and Harris, 1967). For semisolid or solid medium, 0.17% agarose or 1% noble agar was added, respectively. Bacterial cell number was determined using a Petroff-Hausser counting chamber under darkfield microscopy. Bacterial cultures of passage four or less from freezer stocks were used for all experiments; pathogenic strains did not exceed passage eight since isolation from hamsters.

### *E. coli* strains, cloning, and protein purification

*E. coli* strains were grown at 30°C in Luria-Bertani (LB) broth or agar. Strains producing recombinant adhesins and the method for protein purification were previously described (Evangelista et al., 2014b). Proteins were loaded onto SDS-PAGE containing 0.5% 2,2,2-trichloroethanol and imaged by the stain-free method on a ChemiDoc (Bio-Rad) or silver stained.

Plasmid pMaGro (pMaOri containing the *groES* promoter driving expression from the multiple cloning site), and *E. coli* strains π1 (Δ*thyA*) and β2163 (Δ*dapA*) were gifts from Mathieu Picardeau (Institut Pasteur, Paris, France) (Pappas et al., 2015; Gaultney et al., 2020). Primers were designed to amplify the genes encoding LIC11574 and LIC13411 containing overhangs compatible for cloning in the pMaGro plasmid (**Supplementary Table 1**). Genes were amplified from *L. interrogans* sv. Copenhageni genomic DNA, and the PCR products were digested to create cohesive ends. Fragments were cloned in pMaGro digested with the same enzymes and transformed into *E. coli* π1 grown in the presence of thymidine. Plasmids were purified using Promega Wizard Plus SV Minipreps DNA Purification Systems (Promega, Cat. No. A1460) and transformed into *E. coli* β2163 grown in the presence of diaminopimelic acid (DAP). Plasmids harbored in *E. coli* β2163 were conjugated with *L. biflexa* sv. Patoc as described previously (Picardeau, 2008). Sanger sequencing was performed at each step using primers described in **Supplementary Table 1** to confirm correct insertion and maintenance of DNA sequences.

### Ethics statement for animal use

All animals were housed according to institutional guidelines and allowed food and water *ad libitum*. The Medical College of Wisconsin Institutional Animal Care and Use Committee approved all work with animals.

### Production of mouse sera and affinity purification

Mice were immunized similarly to that previously described (Evangelista et al., 2014b). Briefly, five 5-week-old female BALB/c mice were immunized intraperitoneally with recombinant MBP fusions to each adhesin (LIC11574, LIC13411, and control β-galactosidase) or Imject Alum alone (Thermo Scientific, Cat. No. 77161). The initial immunization dose was 100 µg protein in 100 µl phosphate-buffered saline (PBS) mixed with 100 µl Imject Alum. The mice were boosted three times with 50 µg protein in 100 µl PBS mixed with 100 µl Imject Alum at 14-day intervals. Mice were bled by cardiac puncture, and sera were prepared from the collected blood. No reactivity of control sera from alum or MBP-β-galactosidase immunized mice was seen against leptospiral adhesins.

Affinity purification of mouse sera was performed to remove MBP-cross reacting antibodies. MBP-LIC11574 and MBP-LIC13411 were digested with Factor Xa (New England Biolabs, Cat. No. P8010) according to the manufacturer’s instructions. Products that equated to 5 µg undigested MBP-adhesin were loaded in a single large well on SDS-PAGE and transferred to a PVDF membrane. The membrane was blocked with 1% BSA in PBS for 1 h at room temperature and probed overnight at 4°C in blocking buffer with a 1:2,500 dilution of pooled mouse sera for each adhesin. The sides of each membrane were cut off and washed three times with PBS, probed with a 1:10,000 dilution of goat anti-mouse secondary conjugated to alkaline phosphatase (Promega, Cat. No. S372B), and developed with Western Blue^®^ (Promega, Cat. No. S3841) according to the manufacturer’s instructions. Membrane pieces were aligned with the original membrane and used to identify the band corresponding to the adhesin lacking an MBP tag. The unstained piece of the membrane was cut to isolate the band of interest and washed three times with PBS. Each strip was eluted three times with 300 µl of elution buffer (100 mM glycine, 150 mM NaCl, pH 2.4) for 30 s at RT followed by neutralization with 30 µl of 1 M Tris pH 8.5. All three elutions were combined and concentrated approximately 15-fold with Amicon^®^ Ultra Centrifugal Filters (MilliporeSigma, Cat. No. UFC201024). An antibiotic mixture was added to preserve purified antibody stocks (20 µg/ml fosfomycin; 50 µg/ml rifampicin; 5 µg/ml amphotericin B).

Affinity-purified antibodies were assayed against 10-fold dilutions of digested MBP-adhesins. All immunoblots were performed by incubating in blocking buffer for 1 h at room temperature or overnight at 4°C, probing with a primary antibody in blocking buffer for 1 h at room temperature or overnight at 4°C, washing three times, probing with a secondary antibody in blocking buffer for 1 h at room temperature, washing three times, and developing using an appropriate reagent. Antibodies and buffers used are described in **Supplementary Table 2**. All images were obtained on a ChemiDoc (Bio-Rad).

### Cellular fractionation of *Leptospira*

Cellular localization of expressed adhesins was determined by Triton-X 114 fractionation as previously described (Evangelista et al., 2014b). The following modifications were made: Protease Inhibitor Cocktail (MilliporeSigma, Cat. No. P8849) was used instead of adding individual protease inhibitors. Whole-cell lysate (WCL) was harvested by washing bacteria once in PBS followed by resuspending at a concentration of 8 × 10^10^ cells/ml final sample buffer (62.5 mM Tris–HCL pH 6.8; 10% glycerol; 2% SDS; protease inhibitor cocktail). Phase separation and washes were performed by cooling the samples overnight at 4°C, warming them to 37°C for at least 1 h, and centrifuging at 3,400 × g for 30 min in a volume of 10 ml or less to separate aqueous (AQ) and detergent-rich hydrophobic phase (DET). The AQ and DET phases were washed two times as described previously prior to acetone precipitation.

To identify adhesins in each fraction, SDS-PAGE was run by standard protocols loading 4 × 10^8^ cell equivalents per well. Gels were silver stained or immunoblots were performed as described above with reagents shown in **Supplementary Table 2**.

### Human dermal microvascular endothelial cells, growth, and treatments

Human dermal microvascular endothelial cells (HMECs) were obtained from ATCC (CRL-3243) and cultured as described previously in MCDB medium (Sato and Coburn, 2017). Cells at passage 17 or less were used for protein-binding experiments; cells at passage six or less were used for *Leptospira* infection and adhesion experiments. Due to availability at the time of experiments, cells of different passages were used as a new aliquot of cells was received prior to performing the infection and adhesion experiments.

For protein binding and bacterial infections, HMECs were grown on coverslips placed in 12-well plates. Cells were seeded at a density of 4.8 × 10^5^ cells/well and allowed to grow for 2 days. Cells were monitored by brightfield microscopy until confluent. Cells were then washed twice with PBS. For purified protein treatments, protein stocks were diluted to 1 µM in cell culture medium. Five hundred microliters of medium containing appropriate protein was applied to cells and incubated for 1 h. For cell infections with *Leptospira* spp., leptospires were grown to late exponential phase (approximately 1–5 × 10^8^ cells/ml). Cells were infected with 1.38 × 10^7^ leptospires, equating to an MOI of ~20, and incubated for 24 or 30 h.

For all experiments, unbound protein or bacteria was washed away with PBS. Treated cells were fixed with 2% paraformaldehyde in PBS for 15 min at room temperature and washed twice with TBS. Coverslips were blocked with 3% BSA in PBS for 1 h. Antibodies and buffers used are described in **Supplementary Table 3**. Primary antibodies were diluted in blocking buffer and applied to coverslips for 1 h with rocking at room temperature, washed three times with blocking buffer, and then secondary antibodies were added in blocking buffer for 1 h. Coverslips were washed twice with blocking buffer, then washed twice with PBS. Coverslips were mounted on glass slides using ProLong Diamond Antifade Mountant with DAPI (Invitrogen, Cat. No. P36962), cured in the dark overnight, and sealed with nail polish before imaging.

VE-cadherin was stained with an AF488 secondary, except when imaged in conjunction with pathogenic leptospires in which case the secondary for VE-cadherin was conjugated to AF568. The resulting images were artificially colored green (VE-cadherin) or red (pathogenic *Leptospira*) to remain consistent with other images.

### Immunofluorescence microscopy and quantification

All images were acquired on a Nikon Eclipse Ti-U inverted microscope equipped with a CoolSNAP ES2 CCD camera (Photometrics) and a multifluorescent Sedat Quat ET filter set (multichroic splitter, Chroma) using the 20× Plan Apo objective lens (N.A. 0.75, Nikon, Melville). NIS-Elements software (Nikon) was used for acquisition, processing, and analysis.

Mean intensities across each field for each channel were determined using ImageJ and exported to Microsoft Excel for analysis and normalization. For determination of VE-cadherin at cell junctions, automated analysis was performed in NIS-Elements. A binary mask was created in NIS-Elements that encompasses VE-cadherin staining at intercellular junctions. Binary mask parameters were subjectively determined from viewing hundreds of images and used to objectively quantify VE-cadherin staining in all images across numerous days. Each day the mask was evaluated to ensure proper identification of junctional VE-cadherin, adjusted as necessary, and all data analyzed with any change in parameters until final conditions were determined as shown in **Supplementary Figure 1**. To account for day to day variation in signal, the intensity of signal quantified was adjusted to encompass pixels with intensity greater than two times the mean of cells not treated with protein or infected with bacteria (minimizing background which may differ between experiments). The binary area was determined on each image using the binary mask in NIS-Elements and exported to Microsoft Excel for analysis and normalization.

### Collection of endothelial cell lysate and immunoblots for VE-cadherin

Cells were seeded and treated as described above. After removing unbound leptospires, cells were incubated for 5 min at room temperature with 300 µl of 0.05% Triton X-100 and 10 mM EDTA in PBS containing Protease Inhibitor Cocktail (MilliporeSigma, Cat. No. P8849). Cells were scraped and collected. Incubation and scraping were repeated with an additional 100 µl of buffer. Cells were disrupted using a Sonicator 3000 (Misonix). Immunoblots were performed as described above with reagents shown in **Supplementary Table 2**. Densitometry was performed in Image Lab 6.1 (Bio-Rad).

### Adhesion assay and qPCR quantification

To quantify bacterial adhesion to human cells, HMECs were seeded at 4.4 × 10^5^ cells/well in 200 µl in a 96-well tissue culture plate in triplicate for each condition to be tested and grown to post-confluence for 3 days. To prepare plate for infection, 50 µl of medium was removed from each well.

VE-cadherin binding was performed as previously described with some modification (Evangelista et al., 2014a). VE-cadherin was plated at one concentration of 0.1 μM. Protease Inhibitor Cocktail (MilliporeSigma, Cat. No. P8849) was used instead of individual protease inhibitors. Blocking and binding were performed in serum-free MCDB complete medium with 1% BSA. After washing the plate with HBSC (25 mM HEPES pH 7.8, 150 mM NaCl, 1 mM MnCl_2_, 1 mM MgCl_2_, 0.25 mM CaCl_2_) three times, bacteria were added.

For all adhesion assays, leptospires were grown to late exponential phase (approximately 1–5 × 10^8^ cells/ml), pelleted at 7,500 × g for 20 min, and resuspended in MCDB complete medium for cell binding or serum-free MCDB complete medium with 1% BSA for VE-cadherin binding. Bacteria were diluted to the appropriate density, and cells were infected with 50 µl of each bacterial strain containing 3.0 × 10^7^ leptospires/ml equating to an MOI of ~20 for cell-binding experiments, or 50 µl of each strain at 7 × 10^6^ leptospires/ml for VE-cadherin binding. As a control, 50 µl medium alone was added to appropriate wells. The plate was centrifuged at 670 × g for 20 min, followed by incubation for 1 h at 37°C under 5% CO_2_. Unbound bacteria were washed away three times with 200 µl HBSC and wells left empty for DNA extraction. Fifty microliters of inoculum was added to additional wells to quantify bacteria added.

Bacterial DNA was harvested using the DNeasy Blood & Tissue Kit (Qiagen, Cat. No. 69504). To isolate DNA from binding wells, 55 µl sample lysis solution (1 part PBS, 1 part Buffer AL, 1/10-part Proteinase K, and 1/50 parts RNase A) was added to each empty well. In addition, 55 µl inoculum lysis solution (1 part buffer AL, 1/10-part Proteinase K, 1/50-part RNase A) was added to each inoculum well. The plate was incubated at 56°C with shaking for 1 h. Triplicate samples of each condition were combined into one collection tube and purification of DNA proceeded according to the manufacturer’s instructions.

Quantitative polymerase chain reaction (qPCR) was performed using QuantiFast SYBR Green PCR Kit (Qiagen, Cat. No. 204057) using primers for *Leptospira* 16srRNA (**Supplementary Table 1**) and conditions described in an article published in this issue of *Frontiers in Cellular and Infection Microbiology* (Surdel et al., 2022). Briefly, each DNA sample was quantified in triplicate or greater on a Bio-Rad CFX96 cycler. Standard curves were created using genomic DNA isolated from *L. biflexa* sv. Patoc and *L. interrogans* sv. Manilae using the Wizard^®^ Genomic DNA Purification Kit (Promega, Cat. No. A1120), converting mass of DNA in each standard to genomes per reaction using published genome sizes (reviewed in Ko et al., 2009). Samples from the adhesion assay were quantified and fit to the standard curve in Bio-Rad CFX Manager to determine starting quantities in each reaction. Data were exported to Microsoft Excel for further analysis (**Supplementary Tables 4, 5**). Genomes bound per well were compared to the average inoculum for each strain quantified by the qPCR replicates to determine the percent of inoculum that bound for each strain. Due to day-to-day variation in binding, each set of technical replicates was averaged and normalized to the *L. biflexa* pMaGro control strain before being combined with the other independent experiments.

### Short-term mouse infection model

Mouse experiments were performed and samples harvested after 1 h of incubation, as described in an article published in this issue of *Frontiers in Cellular and Infection Microbiology* (Surdel et al., 2022).

### Statistical analysis

All data were graphed, and statistical analyses were performed in GraphPad Prism. All data were analyzed by comparing experimental conditions to the control condition noted in the figure legend for each experiment. Immunofluorescence, cell adhesion, and VE-cadherin adhesion data were analyzed by ordinary one-way ANOVA, correcting for multiple comparisons with the Dunnett test. Data generated from mouse infection experiments were analyzed by Kruskal–Wallis test, correcting for multiple comparisons by controlling the false discovery rate using the method of Benjamini, Krieger, and Yekutieli.

## Results

### *L. interrogans* disrupts VE-cadherin localized to adherens junctions

To determine the effect of *Leptospira* spp. on VE-cadherin, a binary mask was created to identify VE-cadherin located within AJs when imaged by IF microscopy (**Supplementary Figure 1**). This mask was created after viewing hundreds of images and adjusting to ensure all areas identified by the mask were those of VE-cadherin located at junctional complexes. This method allows for accurate quantification of VE-cadherin while avoiding background staining and is therefore independent of day to day variability in signal intensity during image acquisition. The area covered by pixels with intensities that meet the parameters set is then quantified; therefore, the result indicates VE-cadherin located in AJs (**Supplementary Figure 1**).

**Figure 1:**
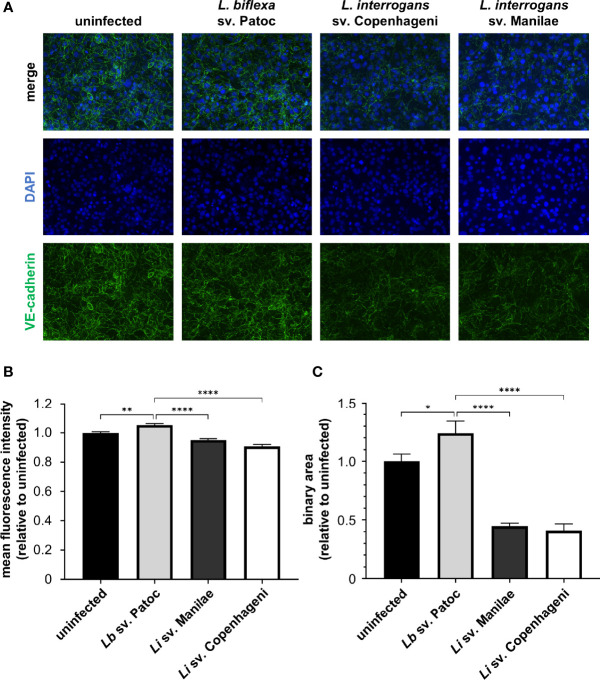
Pathogenic *Leptospira* disrupt VE-cadherin staining in adherens junctions (AJs). HMECs were grown to confluence on glass coverslips and infected at an MOI of 20 for 24 h. Cells were fixed, stained for VE-cadherin (green), and mounted with Prolong Diamond Antifade Mountant with DAPI (blue). **(A)** Representative images highlight the change in VE-cadherin staining upon infection with various strains of *Leptospira*. **(B)** Mean fluorescence intensity (m.f.i.) was quantified. Minimal decreases in VE-cadherin are seen upon treatment with pathogenic *Leptospira*. **(C)** Binary area of VE-cadherin was determined. Pathogenic *Leptospira* quantitatively decrease VE-cadherin localized in AJs. Data represent three independent experiments; 10 images per slide were taken and analyzed quantitatively for each experiment. Mean ± SEM is plotted. Each column is compared to the *Lb* sv. Patoc control. *denotes *p* ≤ 0.05, **denotes *p* ≤ 0.01, ****denotes *p* ≤ 0.0001.

Previously, it has been shown that *L. interrogans* sv. Copenhageni decreases overall VE-cadherin staining (Sato and Coburn, 2017). To determine if these changes are due to reductions in VE-cadherin within AJs, HMECs were infected with *L. interrogans* svv. Copenhageni and Manilae as well as *L. biflexa* sv. Patoc as a non-pathogenic control at an MOI of 20 and allowed to proceed for 24 h. Both pathogenic strains of *Leptospira* qualitatively disrupt junctional VE-cadherin when compared to uninfected controls, suggesting that VE-cadherin disruption is a conserved effect of pathogenic *Leptospira* (**Figure 1A**). This qualitative disruption corresponds to a slight, yet significant, decrease in overall VE-cadherin when measured by mean fluorescence intensity (m.f.i.) of cells infected with pathogenic *L. interrogans* in comparison with uninfected or non-pathogenic-infected controls (**Figure 1B**). To determine if this is due to changes in junctional VE-cadherin, the binary mask described above was applied. Pathogenic *Leptospira* reduce junctional VE-cadherin, whereas non-pathogenic *Leptospira* do not alter VE-cadherin staining within AJs (**Figure 1C**). Quantifying VE-cadherin by binary area has a larger dynamic range, and therefore more subtle changes in VE-cadherin can be determined. Taken together, this implies that VE-cadherin disruption could be a conserved activity induced by multiple pathogenic *Leptospira* isolates.

### VE-cadherin is not degraded or phosphorylated

VE-cadherin localization is regulated through numerous mechanisms. One way in which VE-cadherin levels change is through endocytosis and degradation (Su and Kowalczyk, 2017). Importantly, the changes in VE-cadherin seen in IF microscopy indicate a change in surface-exposed VE-cadherin, as cells were not permeabilized prior to staining. Therefore, to determine if pathogenic *Leptospira* were causing VE-cadherin endocytosis and degradation, immunoblots were performed on cell lysates after infection. No strain of *Leptospira* induces detectible changes in total VE-cadherin levels as measured by immunoblotting, nor do they lead to the accumulation of any degradation products, suggesting that VE-cadherin does not undergo degradation (**Supplementary Figure 2**).

VE-cadherin function can also be regulated by phosphorylation (Potter et al., 2005; Allingham et al., 2007; Wallez et al., 2007; Turowski et al., 2008; Adam et al., 2010; Orsenigo et al., 2012; Wessel et al., 2014; Sidibe and Imhof, 2014). Y658 and Y731 are two residues of VE-cadherin that are known to be involved in phosphorylation-dependent regulation of vascular permeability and have commercially available phospho-specific antibodies. Cell lysates were probed with antibodies to phosphorylated versions of Y658 and Y731; however, no change in phosphorylation status of these residues was seen upon infection with any *Leptospira* strain (**Supplementary Figure 2**). In summary, these data indicate that infection with pathogenic *Leptospira* alters VE-cadherin localization, although does not affect overall cadherin levels or phosphorylation status.

### LIC11574 and LIC13411 bind to endothelial cells

**Figure 2:**
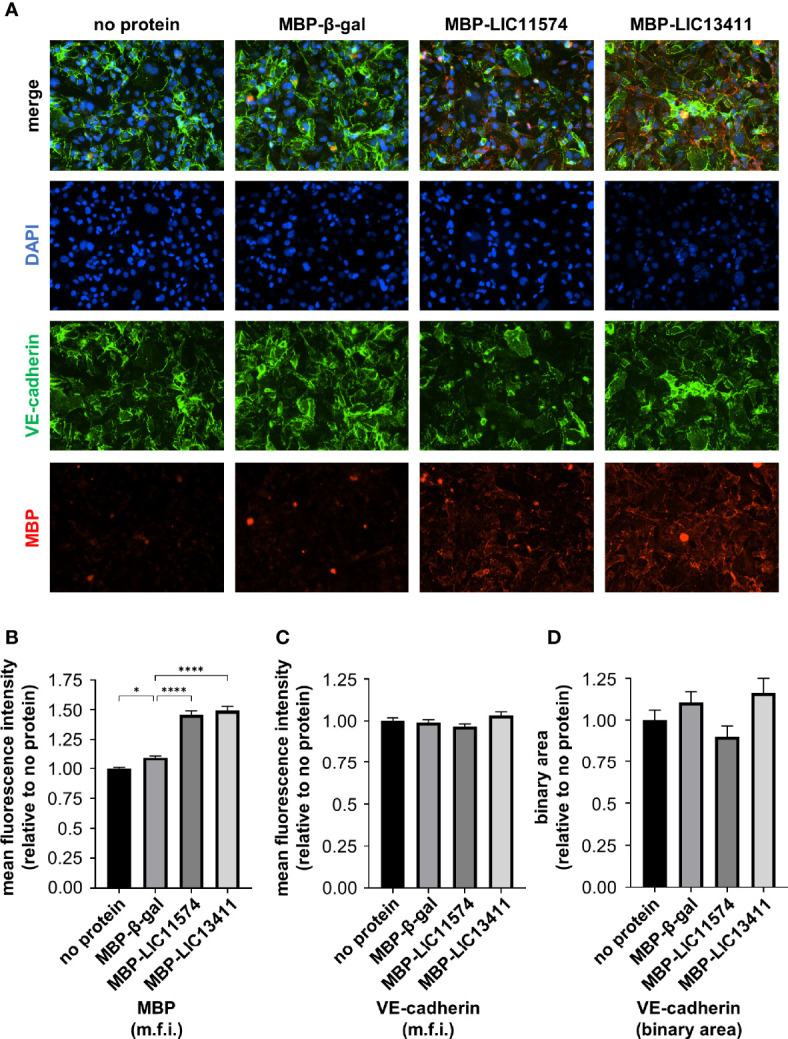
Adhesins from *L. interrogans* bind endothelial cells. HMECs were grown to confluence on glass slides and treated with purified MBP-adhesin fusions for 1 h. Slides were stained for VE-cadherin (green) and MBP (red), and mounted with Prolong Diamond Antifade Mountant with DAPI (blue). **(A)** MBP-adhesin fusions bind to endothelial cells in a pattern reminiscent of VE-cadherin; however, little overlap is seen. **(B)** M.f.i. of MBP was quantified. MBP-adhesin fusions bind to cells significantly more than cells treated with MBP-β-gal control and significantly over background signal from untreated cells. **(C)** M.f.i. of VE-cadherin was quantified. Total VE-cadherin staining does not change with MBP-adhesin treatment. **(D)** Binary areas of VE-cadherin in AJs were determined. No change in VE-cadherin localized to AJs is seen with purified MBP-adhesin treatment. Data represent three independent experiments; 10 images per slide were taken and analyzed quantitatively for each experiment. Mean ± SEM is plotted. Each column is compared to the MBP-β-gal control. *denotes *p* ≤ 0.05, ****denotes *p* ≤ 0.0001.

Previously, our laboratory identified two candidate adhesins, LIC11574 and LIC13411, that bind VE-cadherin *in vitro* (Evangelista et al., 2014b). To investigate whether the candidate adhesins bind HMECs, colocalize with VE-cadherin, and disrupt VE-cadherin staining, MBP-adhesin fusion proteins were recombinantly produced in and purified from *E. coli* (**Supplementary Figure 3**). Confluent HMECs were treated with MBP-adhesin fusions for 1 h. Adhesins attach to endothelial cells in a pattern reminiscent of VE-cadherin; however, little colocalization is seen (**Figure 2A**). Importantly, MBP-adhesin fusions bind significantly better to endothelial cells than does MBP-β-gal when measured by m.f.i., suggesting specificity of purified adhesins to endothelial cell factors (**Figure 2B**). Neither MBP-LIC11574 nor MBP-LIC13411 alters overall VE-cadherin staining when measured by m.f.i. (**Figure 2C**). In addition, neither candidate adhesin induces a change in localization of VE-cadherin away from AJs when using the binary mask (**Figure 2D**). In summary, the adhesins bind endothelial cells in a pattern similar to VE-cadherin staining; however, the candidate adhesins alone are insufficient to disrupt VE-cadherin localization.

### Heterologous production of candidate adhesins in *L. biflexa*

It is possible that the adhesins require other bacterial factors in order to alter VE-cadherin localization. Therefore, the genes encoding LIC11574 and LIC13411 were cloned in the pMaOri plasmid under the control of the *groES* promoter (Pappas et al., 2015; Gaultney et al., 2020). The resulting plasmid was conjugated into *L. biflexa* sv. Patoc, creating gain-of-function (GOF) strains producing either LIC11574 or LIC13411.

Previous research has shown that the proteins LIC11574 and LIC13411 localize to the outer membrane in *L. interrogans* sv. Copenhageni (Evangelista et al., 2014b). To determine if the proteins are produced in the GOF strains, and whether they localize to the outer membrane, Triton X-114 fractionation was performed. Antibodies to proteins known to localize to cellular compartments have been developed only against pathogen homologs, thereby limiting our ability to identify all of these proteins in non-pathogenic strains due, in some cases, to low sequence conservation. In parallel to the GOF strains, we performed fractionation of *L. interrogans* sv. Manilae to serve as a control.

**Figure 3:**
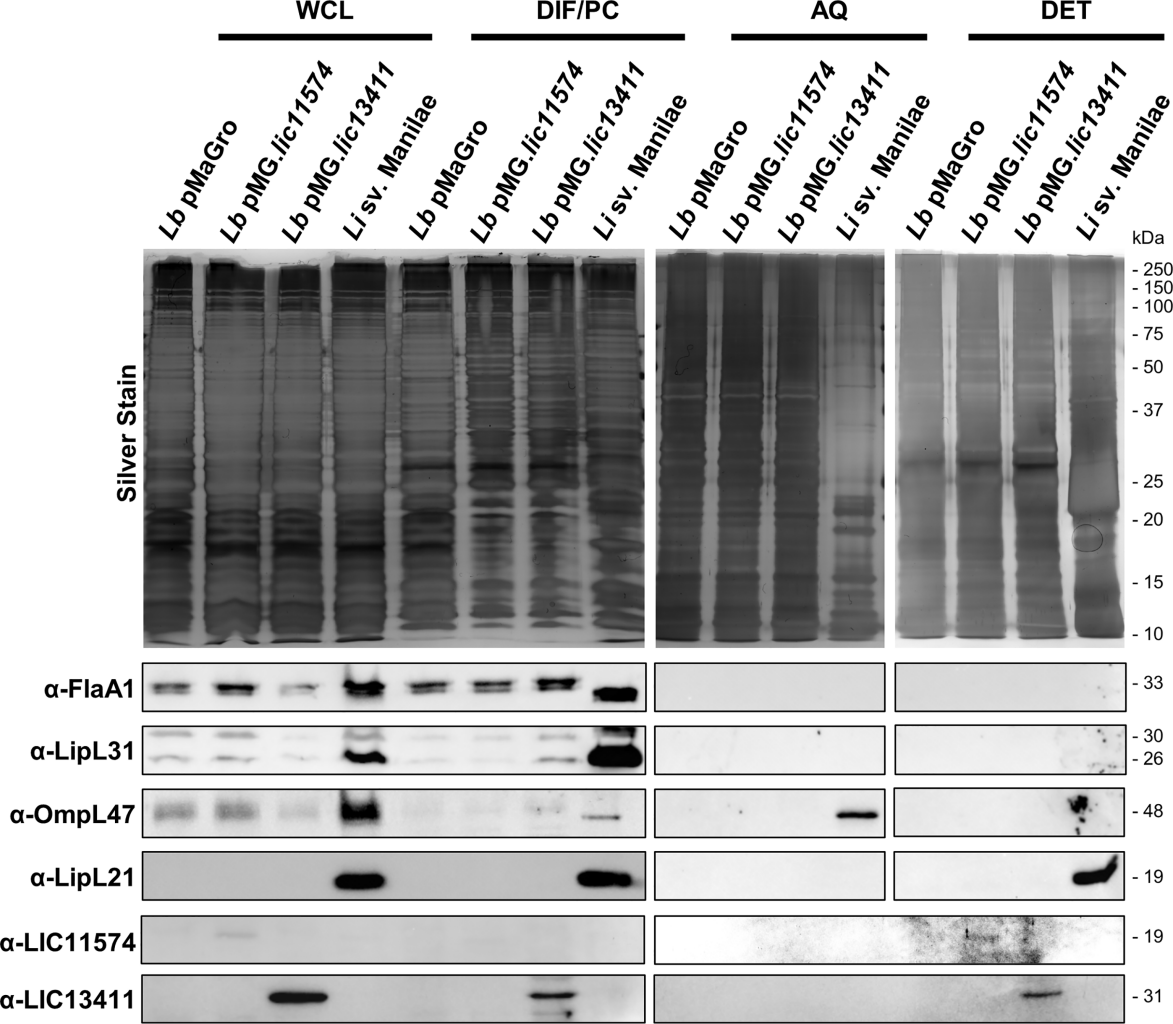
Adhesins produced in *L. biflexa* localize to the outer membrane. *Leptospira* strains were fractionated using Triton X-114. Samples corresponding to 4 × 10^8^ *Leptospira* cell equivalents were loaded in each lane corresponding to whole-cell lysate (WCL), detergent-insoluble fraction/protoplasmic cylinder (DIF/PC), aqueous (AQ), and detergent (DET) fractions. A silver stain was performed on each fraction to show protein loaded (top). A bubble is present in the silver stain of the DET fraction of *L. interrogans* sv. Manilae. Immunoblots were performed using sera against periplasmic protein flagellin A1 (FlaA1), inner membrane protein LipL31, outer membrane proteins OmpL47 and LipL21, and affinity-purified mouse sera against LIC11574 and LIC13411. Both candidate adhesins are produced in the gain-of-function (GOF) strains. Candidate adhesin LIC11574 localizes to the DET fraction; however, to identify the band, the brightness and contrast of the image were increased 40% and 60%, respectively. LIC13411 localizes to the DIF/PC and DET phases.

As expected, all control proteins were identified in the WCL fraction (**Figure 3**). Flagellin protein A1 (FlaA1) localizes to the detergent insoluble fraction/protoplasmic cylinder (DIF/PC) in all strains, consistent with it being a periplasmic protein. Known inner membrane protein LipL31 also localizes to the DIF/PC in both pathogenic and non-pathogenic strains. Outer membrane proteins OmpL47 and LipL21 fractionated to the aqueous (AQ) and detergent (DET) fractions, respectively, but were only identified significantly in the pathogen lysate. The localization of proteins seen is consistent with previous studies and indicates successful fractionation (Evangelista et al., 2014b).

In order to identify the candidate adhesins by immunoblot, mouse anti-sera were produced against each protein. Mice were immunized with MBP-LIC11574 and MBP-LIC13411, and the resulting sera were affinity purified to each candidate adhesin lacking the MBP tag. To determine a general affinity of these antibodies, dilutions of digested MBP-adhesin constructs were probed with their respective serum. The affinity-purified α-LIC11574 serum was able to detect LIC11574 lacking the MBP tag after digestion of 40 ng of MBP-LIC11574, whereas the affinity-purified α-LIC13411 serum was only able to identify LIC13411 from digestion of 400 ng of MBP-LIC13411 (**Supplementary Figure 4**). The affinity-purified sera were then used to probe for LIC11574 and LIC13411 in the fractionated lysates. Both candidate adhesins can be seen in their respective GOF strain WCL, while being absent from a strain harboring the empty pMaGro plasmid (**Figure 3**). Both candidate adhesins localize to the DET fraction, consistent with previous fractionation of *L. interrogans* sv. Copenhageni (Evangelista et al., 2014b). Of note, the band corresponding to LIC11574 is extremely faint, and taken in combination with the fact that the antibody is more sensitive in detection of recombinant protein than that for LIC13411, this suggests that very low levels of LIC11574 are being produced in the GOF strain. Interestingly, neither adhesin was identified in *L. interrogans* sv. Manilae, implying low or absent levels of these adhesins in this strain. Taken together, these data indicate that the GOF strains produce and localize the candidate adhesins to the outer membrane.

### GOF strains do not disrupt VE-cadherin localization in AJs

**Figure 4:**
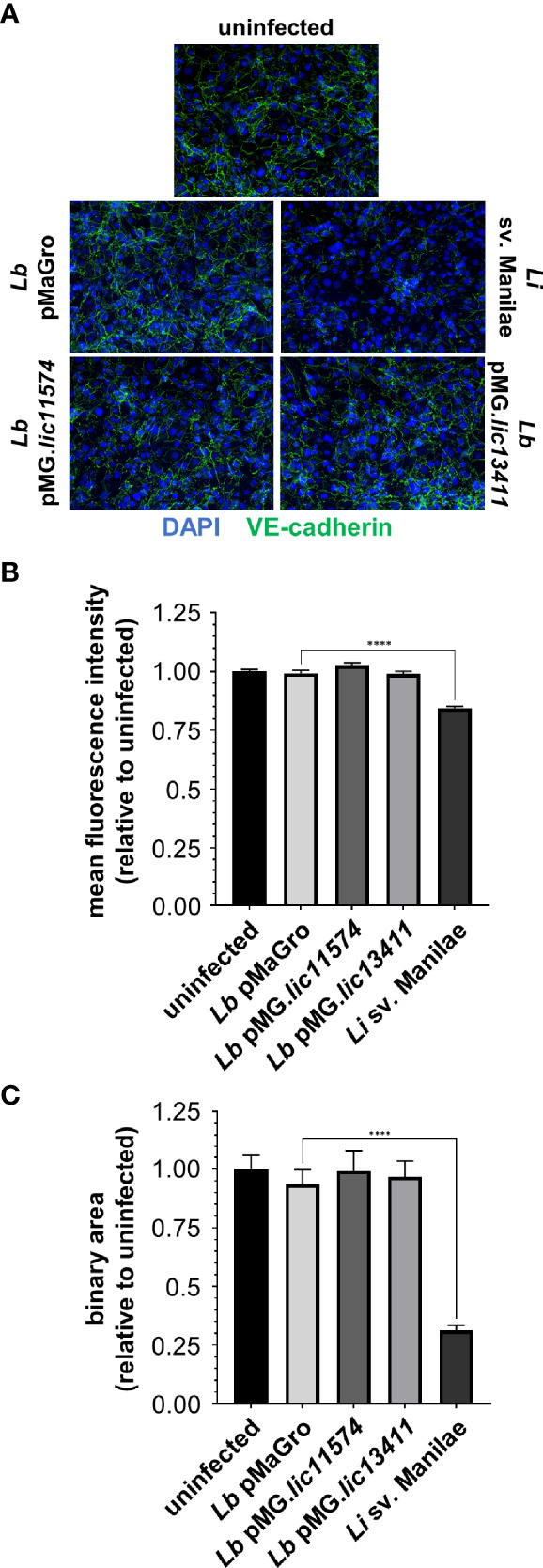
*L. biflexa* producing LIC11574 and LIC13411 do not disrupt VE-cadherin staining. HMECs were grown to confluence on glass coverslips and infected at an MOI of 20 for 24 h. Cells were fixed, stained for VE-cadherin (green), and mounted with Prolong Diamond Antifade Mountant with DAPI (blue). **(A)** Representative images are shown highlighting the change in VE-cadherin staining upon infection with various strains of *Leptospira* spp. Pathogenic *Leptospira* disrupts VE-cadherin localized in AJs, whereas the non-pathogen and GOF strains do not. **(B, C)** M.f.i. and binary area were quantified. No change in total VE-cadherin or VE-cadherin associated with AJs is seen upon treatment with non-pathogenic or GOF strains, whereas pathogenic *Leptospira* disrupt VE-cadherin localization. Data represent two independent experiments; 10 images per slide were taken and analyzed quantitatively for each experiment. Mean ± SEM is plotted. Each column is compared to the *L. biflexa* pMaGro control. **** denotes *p* ≤ 0.0001.

We hypothesized that production of the candidate adhesin in a non-pathogen would lead to alteration of VE-cadherin localization. Upon infection with the GOF strains, there is no qualitative change in VE-cadherin staining, suggesting no effect of the adhesin-producing strains (**Figure 4A**). VE-cadherin was quantified by m.f.i. and binary mask, and no change is seen in VE-cadherin upon infection with these strains (**Figures 4B, C**). As a control, *L. interrogans* sv. Manilae was used and led to a significant disruption of VE-cadherin staining (**Figure 4**). Therefore, the candidate adhesins are not sufficient to induce VE-cadherin re-localization, and other pathogenic bacterial factors are likely necessary to disrupt VE-cadherin localized to AJs.

### Adhesin LIC13411 produced in *L. biflexa* increases cell and VE-cadherin binding

**Figure 5:**
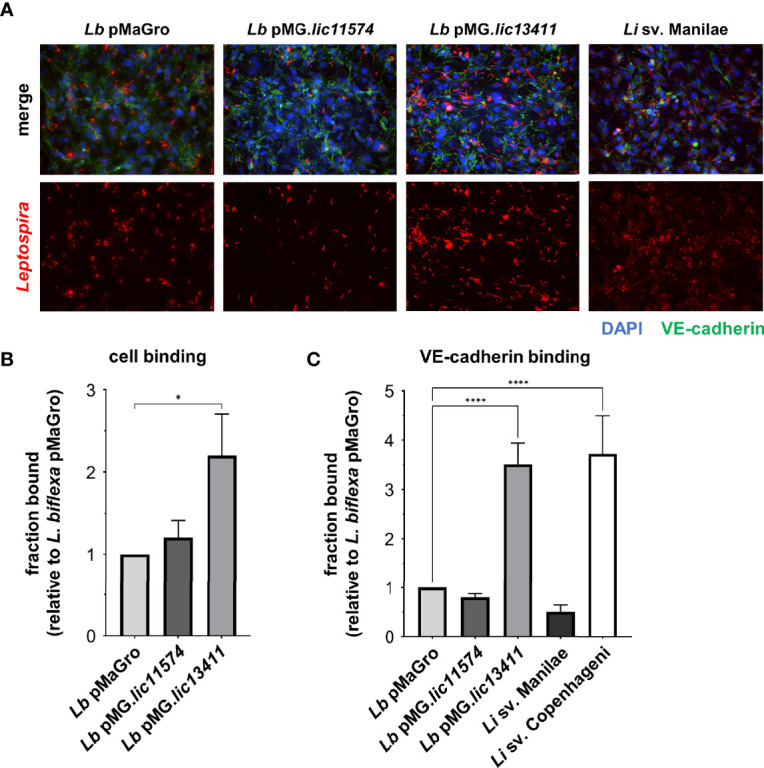
LIC13411 production in *L. biflexa* increases binding to HMECs and VE-cadherin. **(A)** HMECs were grown to confluence on glass coverslips and infected at an MOI of 20 for 30 h. Cells were fixed, stained for VE-cadherin (green) and *Leptospira* (red), and mounted with Prolong Diamond Antifade Mountant with DAPI (blue). Representative images were selected by an unbiased, blinded observer. *L. biflexa* pMG.*lic13411* binds to HMEC cells more than *L. biflexa* pMaGro. **(B)** HMECs were grown to post-confluence, *Leptospira* strains were added at an MOI of 20, incubated for 1 h, and qPCR was performed to quantify bound bacteria. *L. biflexa* pMG.*lic13411* bind to HMECs significantly more than *L. biflexa* pMaGro. Data represent four independent experiments. **(C)** VE-cadherin was plated, 3.5 × 10^5^ leptospires were added to each well, incubated for 1 h, and qPCR was performed to quantify bound bacteria. *L. biflexa* pMG.*lic13411* bound to VE-cadherin significantly more than *L. biflexa* pMaGro and bound similarly to VE-cadherin as did pathogenic *L. interrogans* sv. Copenhageni. Data represent five independent experiments, three of which utilized *L. interrogans* sv. Copenhageni. For all data, technical replicates each day were averaged. Mean ± SEM is plotted. Each column is compared to the *L. biflexa* pMaGro control. *denotes *p* ≤ 0.05, ****denotes *p* ≤ 0.0001.

Not all adhesins exhibit multiple functions. The main, and defining, characteristic of an adhesin is its ability to facilitate bacterial binding to host cells and molecules. Confluent HMECs were either left uninfected or infected with the GOF or pathogenic *Leptospira* strains to quantify adhesion. At 30 h postinfection, cells were fixed and stained with antibodies against either non-pathogenic or pathogenic *Leptospira* to identify the GOF strains or *L. interrogans* sv. Manilae, respectively. Considerably higher levels of binding are seen with *L. biflexa* sv. Patoc pMG.*lic13411* than with either *L. biflexa* sv. Patoc pMaGro or pMG.*lic11574* (**Figure 5A**). In addition, *L. interrogans* sv. Manilae shows significant binding (**Figure 5A**). Binding was then quantified by qPCR following incubation of HMECs with *Leptospira*. Consistent with the IF microscopy, binding of *L. biflexa* sv. Patoc pMG*.lic13411* is significantly higher than *L. biflexa* sv. Patoc pMaGro or pMG.*lic11574*, meaning that LIC13411 plays an important role in adhesion of bacteria to endothelial cells (**Figure 5B**; **Supplementary Figure 5**, **Supplementary Tables 4, 5**). In a second approach, VE-cadherin was immobilized and incubated with various strains of bacteria followed by quantification by qPCR to determine the specificity of the adhesin for the known *in vitro* binding partner. *L. biflexa* sv. Patoc pMG*.lic13411* binds significantly better to VE-cadherin than the control strain (**Figure 5C**; **Supplementary Figure 5**, **Supplementary Tables 4, 5**). Importantly, it binds to a similar extent as the pathogenic strain *L. interrogans* sv. Copenhageni, suggesting that LIC13411 is sufficient to promote VE-cadherin binding by a non-pathogen (**Figure 5C**; **Supplementary Figure 5**, **Supplementary Tables 4, 5**). Interestingly, *L. interrogans* sv. Manilae did not bind VE-cadherin. This is consistent with immunoblots performed showing undetectable levels of both adhesins in *L. interrogans* sv. Manilae (**Figure 3**), whereas previous studies have identified the adhesin as present in *L. interrogans* sv. Copenhageni (Evangelista et al., 2014b). Overall, LIC13411 is confirmed to be a leptospiral adhesin facilitating binding to endothelial cells and VE-cadherin.

### LIC13411 production increases bacterial burdens in the lung, liver, kidney, and bladder

**Figure 6:**
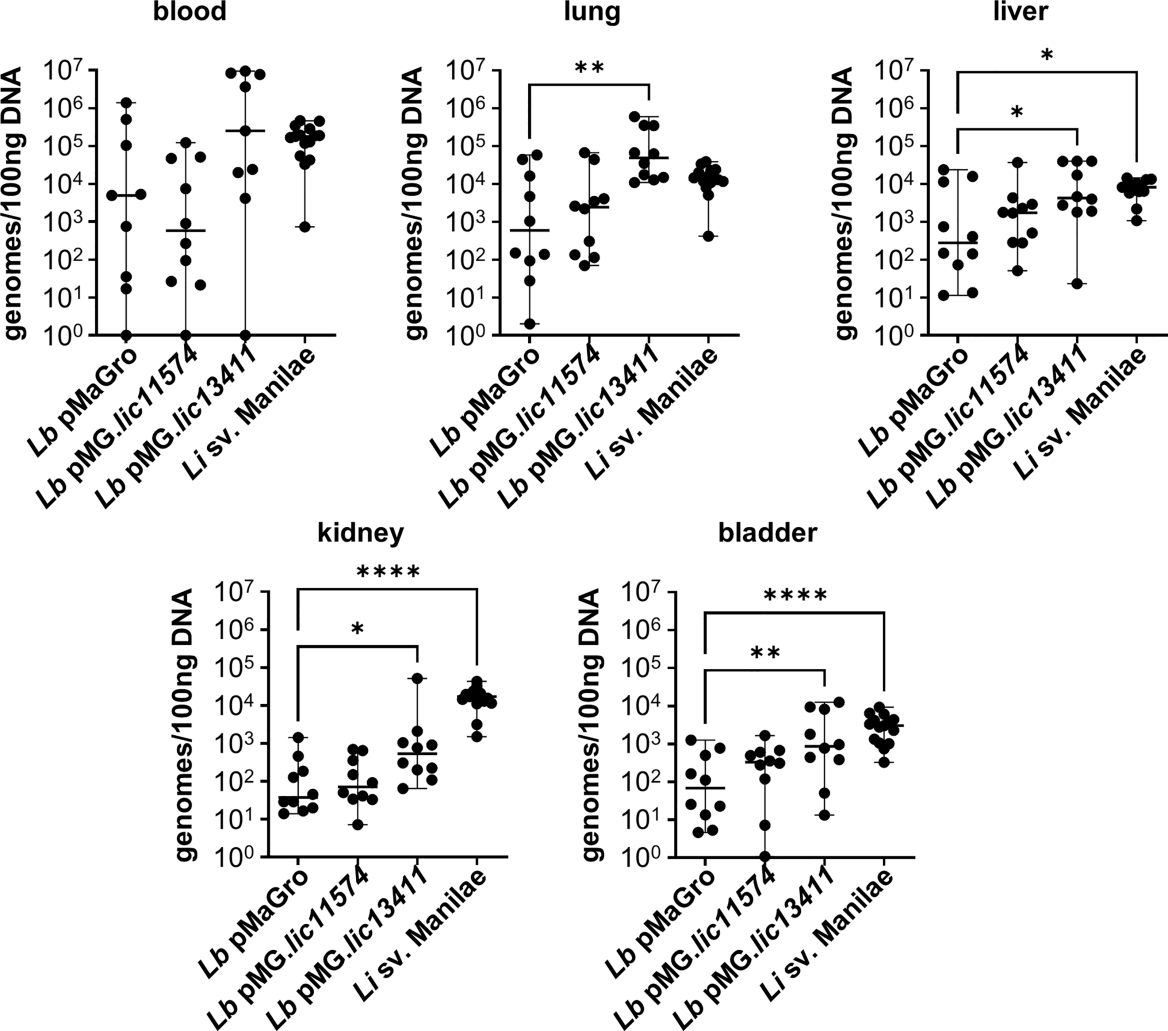
LIC13411 increases bacterial burdens in the lung, liver, kidney, and bladder. Mice were inoculated intravenously in a 1 h model of infection. DNA from organs was harvested and quantified by qPCR. Significantly higher burdens of *L. biflexa* pMG.*lic13411* were found in the lung, liver, kidney, and bladder relative to the non-pathogenic control strain. The burden of *L. biflexa* pMG.*lic13411* in the bladder is similar to the burden of pathogenic *L. interrogans* sv. Manilae. Data represent a total of 10 mice per group (except for blood samples with *L. biflexa* pMaGro and *L. biflexa* pMG.*lic13411* where n = 9), performed as two independent experiments per strain. Data for *L. interrogans* sv. Manilae originally appears in an article published in this issue of *Frontiers in Cellular and Infection Microbiology* and represents three independent experiments per strain with 15 mice per group (except for liver where n = 13) (Surdel et al., 2022). Median ± range is plotted. Each group is compared to the *L. biflexa* pMaGro control. *denotes *p* ≤ 0.05, **denotes *p* ≤ 0.01, ****denotes *p* ≤ 0.0001.

Previously, our laboratory has described a short-term model of *Borrelia burgdorferi* infection developed to investigate the role of bacterial adhesins in hematogenous dissemination (Caine and Coburn, 2015). In a manuscript published in this issue of *Frontiers in Cellular and Infection Microbiology*, we describe the adaptation of this method to *Leptospira*. We show that pathogenic *Leptospira* have higher bacterial burdens in the blood, liver, kidney, and bladder, consistent with the kidney being the biologically essential site of colonization for pathogenic *Leptospira*. In addition, detectable burdens of *L. biflexa* were identified in numerous organs, suggesting that this model could be useful in interrogating GOF strains developed in non-pathogens (Surdel et al., 2022). Therefore, mice were inoculated with the GOF strains and organs harvested after 1 h. *L. biflexa* sv. Patoc pMG*.lic13411* showed increased burdens in multiple organs (**Figure 6**). Despite the short-term nature of this model, LIC13411 can facilitate increased adhesion and burdens within the lung, liver, kidney, and bladder. Burdens of *L. biflexa* sv. Patoc pMG*.lic13411* in the kidney are significantly greater than those of *L. biflexa* sv. Patoc pMaGro, identifying LIC13411 as crucial for dissemination to this vital organ in the life cycle of *Leptospira* in nature. Pulmonary hemorrhage is a major sequela of leptospirosis, and LIC13411 additionally facilitates increased bacterial burdens in the lungs, suggesting a role for LIC13411 in tropism to the lung as well (**Figure 6**). Taken together, this work provides the first evidence that LIC13411 facilitates binding of a living bacterium to host components *in vivo* and increases a pathogenic property of a non-pathogen in a mouse model of infection.

## Discussion

Bacterial adherence to host components is generally crucial for infection and pathogenesis, and proteins that mediate bacterial binding to the host are termed adhesins. Our laboratory has previously shown that pathogenic leptospires bind to host cells with greater efficiency than to the extracellular matrix and specifically that candidate adhesins LIC11574 and LIC13411 bind to VE-cadherin *in vitro* (Evangelista et al., 2014a; Evangelista et al., 2014b). Interrogating the specific role of these two candidate adhesins in pathogenesis has been difficult due to the limited genetic tools available in *Leptospira*. Recent advances in *Leptospira* genetics have provided many tools necessary to further dissect pathogenic mechanisms in greater detail (Aviat et al., 2010; Pappas et al., 2015; Gaultney et al., 2020; Fernandes et al., 2021; reviewed in Ko et al., 2009). In the current study, we determined the role of candidate adhesins in the infectivity-promoting properties of pathogenic *Leptospira*.

*L. interrogans* has been shown to decrease overall VE-cadherin staining by IF microcopy (Sato and Coburn, 2017). Using mean fluorescence intensity to quantify VE-cadherin has multiple limitations, including being highly dependent upon background staining which can vary from day to day. In order to optimize quantification of VE-cadherin in IF microscopy, we developed a method for evaluating VE-cadherin located specifically within AJs independent of background signal and day-to-day variation. Using a binary mask, we show that although there is only a slight reduction in overall VE-cadherin signal upon infection of endothelial cells with pathogenic *Leptospira*, the localization of VE-cadherin is significantly disrupted (**Supplementary Figure 1** and **Figure 1**). This altered VE-cadherin signal is not due to degradation or phosphorylation status, but rather points to a model where VE-cadherin is re-localized away from AJs (**Supplementary Figure 2**). Furthermore, VE-cadherin disruption is induced by two widely studied pathogenic *L. interrogans* strains, suggesting a conserved pathogenic mechanism. One hallmark feature of severe leptospirosis is hemorrhage, and disruption of endothelial junctions through alteration of VE-cadherin could explain this disease manifestation. In addition, disruption of endothelial barriers facilitates entry into and exit from the bloodstream during bacterial dissemination and disease.

LIC11574 and LIC13411 from pathogenic *Leptospira* bind VE-cadherin *in vitro* with single-digit nanomolar affinities (Evangelista et al., 2014b). When incubated with endothelial cells, these adhesins bind significantly more than the non-specific control protein β-gal, and the candidate adhesins bind in a pattern reminiscent of VE-cadherin, implying a potential interaction in living cells; however, no overlap between proteins is seen (**Figure 2**). This could be due to numerous factors. An MBP tag was chosen for these constructs for multiple reasons. As the adhesins are outer membrane proteins, solubility was an issue when attempting to create constructs with smaller tags. In addition, MBP is less likely to be folded into the tertiary structure of the protein, allowing access by commercially available antibodies to detect MBP-adhesin fusions by IF microscopy. One disadvantage of using MBP is the large size, and the steric hinderance imparted by the MBP tag could limit access of antibodies to VE-cadherin. Further studies will attempt to determine whether the adhesins and VE-cadherin are interacting in IF experiments.

Regardless, the purified adhesins do not alter VE-cadherin localization to AJs and are therefore not alone sufficient to induce phenotypes seen with pathogenic *Leptospira*. We therefore hypothesized that expression of the adhesin in the context of a living organism (i.e., non-pathogenic *L. biflexa*) would lead to disruption of VE-cadherin localization, as additional bacterial factors are present. Using recently developed genetic tools, we created GOF strains of the non-pathogen *L. biflexa* producing LIC11574 or LIC13411. To confirm that these strains were producing the candidate adhesins, Triton X-114 fractionation and immunoblotting were performed. Importantly, both strains produce their respective adhesins, but to different extents. LIC11574 was barely detectible, despite the affinity-purified mouse antibody having greater sensitivity than that directed against LIC13411 (**Figure 3** and **Supplementary Figure 4**). Therefore, even though the candidate adhesins are under the control of the same strong promoter, little LIC11574 is ultimately produced and localized to the outer membrane, limiting the ability to identify GOF phenotypes in this strain. Future studies will attempt to create a GOF strain of LIC11574 under the control of a different promoter or modified ribosome-binding site in an effort to increase LIC11574 production. In addition, *L. interrogans* sv. Manilae did not produce detectible levels of either adhesin. This is in contrast to previously published data showing that *L. interrogans* sv. Copenhageni does produce detectable levels of the two candidate adhesins, thereby pointing to differential regulation and expression of these genes among pathogenic strains of *Leptospira*, despite identical amino acid sequences of the proteins (Evangelista et al., 2014b). Importantly, growth in laboratory medium does not mimic the natural infection process. Growth of *L. interrogans* sv. Manilae in various conditions may allow us to gain insight into the regulation of LIC13411 production in this pathogenic strain and perhaps point to increased LIC13411 production during the pathogenesis of *Leptospira in vivo*.

To test whether LIC11574 and LIC13411 alter VE-cadherin localization in the context of a living organism, endothelial cells were infected with the GOF strains. Importantly, neither adhesin led to detectable changes in VE-cadherin signal or localization (**Figure 4**). Therefore, the adhesins are not sufficient to disrupt VE-cadherin, even when produced by living leptospires. This points to a much more complex system of VE-cadherin alteration. It is therefore likely that additional pathogen-produced factors are required to induce VE-cadherin re-localization.

Although many adhesins exhibit multiple functions, the defining function of an adhesin is the ability to bind host factors. Using IF microscopy, greater numbers of *L. biflexa* sv. Patoc pMG.*lic13411* bound cell monolayers than did the empty plasmid control strain (**Figure 5A**). To quantify binding of the GOF strains to endothelial cells, qPCR was performed in a similar experiment. LIC13411 production led to significantly higher binding to endothelial cells (**Figure 5B**). Although LIC11574 did not lead to increased binding of cells, it was previously shown that the K_D_ for LIC11574 binding VE-cadherin is 20-fold higher (i.e., lower affinity) than that of LIC13411 (Evangelista et al., 2014b), and LIC11574 production is much lower than that of LIC13411 in the GOF strains (**Figure 3**). It is therefore not surprising that the LIC11574 GOF strain did not lead to increased binding of endothelial cells. Because these adhesins were originally identified to bind VE-cadherin, VE-cadherin was immobilized and binding of GOF strains was assessed. The GOF strain producing LIC13411 bound significantly higher to VE-cadherin than did the control strain, suggesting a specific interaction between LIC13411 and VE-cadherin in a living bacterium (**Figure 5C**). *L. interrogans* sv. Copenhageni bound to VE-cadherin, whereas *L. interrogans* sv. Manilae did not, correlating with the production of LIC13411 by the two strains noted above. Finally, LIC13411 facilitated binding of *L. biflexa* to a similar extent as the pathogenic strain, leading to the conclusion that LIC13411 is sufficient to facilitate binding of non-pathogenic *Leptospira* to VE-cadherin. Taken together, this work has confirmed the role of LIC13411 as a bacterial adhesin. This is the first evidence that LIC13411 can facilitate binding of a living organism to endothelial cells and VE-cadherin. Pathogens contain numerous virulence factors, and as these adhesins are not alone sufficient to disrupt VE-cadherin localization, it is likely that other unidentified factors produced by the pathogens are required and that LIC13411 likely facilitates binding of bacteria to host cells to allow these factors to function.

Previously our laboratory developed a short-term model of infection to interrogate adhesin proteins in *B. burgdorferi*. In a manuscript published in this issue of *Frontiers in Cellular and Infection Microbiology*, we describe the adaptation of this model to *Leptospira* (Surdel et al., 2022). To investigate the roles of the candidate adhesins LIC11574 and LIC13411 *in vivo*, mice were inoculated intravenously with each GOF strain and burdens in numerous organs were quantified by qPCR. Production of LIC13411 led to increased burdens in the lung, liver, kidney, and bladder (**Figure 6**). Pulmonary hemorrhage is a hallmark of leptospirosis, and these data suggest that LIC13411 may facilitate binding and dissemination to the lung. In addition, *Leptospira* must colonize the kidney, and LIC13411 may play a role in dissemination to this organ as well. Furthermore, this phenotype was seen just 1 h after infection, suggesting the importance of LIC13411 in early stages of tissue colonization and potentially pathogenesis. In sum, LIC13411 increases bacterial binding to host cells *in vitro* and promotes binding and increased burdens in multiple organs *in vivo*.

Targeting bacterial adhesion is a promising antibacterial strategy and provides numerous advantages to conventional therapy (Okuda et al., 2010; Huebinger et al., 2016; reviewed in Svensson et al., 2006; Krachler and Orth, 2013; Stones and Krachler, 2016; Asadi et al., 2019). Since targeting adhesion does not change the overall fitness of the organism, it does not impose the same selective pressure leading to resistance as do conventional antibiotics (reviewed in Krachler and Orth, 2013). Interestingly, both LIC11574 and LIC13411 were identified as potential vaccine candidates by other laboratories (Gamberini et al., 2005; Grassmann et al., 2017). In addition, the human humoral response is important for protection from pathogenic *Leptospira* spp., and antibodies to LIC11574 and LIC13411 have been identified in patients who have been exposed to pathogenic *Leptospira* (Evangelista et al., 2014b). Therefore, future studies investigating the ability of anti-adhesin therapy targeting LIC13411 as a potential therapeutic in limiting *Leptospira* infection are of interest.

Prior to this work, our laboratory identified two candidate adhesins, LIC11574 and LIC13411. In this study, we have shown that two distinct pathogenic *L. interrogans* strains disrupt VE-cadherin. Although neither adhesin leads to disruption of VE-cadherin, this study provides the first evidence that LIC13411 facilitates binding of a living bacterium to human cells and VE-cadherin, confirming its role as an adhesin and therefore identifying LIC13411 as a potential therapeutic target. In addition, LIC13411 has been shown to be important in a murine model of infection. Ultimately, this work has provided novel insight into the pathogenic mechanisms *Leptospira* employ and provides the foundation for the development of novel prophylactic and therapeutic strategies.

## Data Availability Statement

The original contributions presented in the study are included in the article/**Supplementary Material**. Further inquiries can be directed to the corresponding author.

## Ethics Statement

The animal study was reviewed and approved by Institutional Animal Care and Use Committee, Medical College of Wisconsin.

## Author Contributions

MS and JC designed the experiments. MS, BH, and PA performed the experiments included in this publication. MS performed the analysis and interpreted the data. MS wrote the manuscript. BH, PA, and JC edited the manuscript. All authors contributed to the article and approved the manuscript for submission.

## Funding

This work was funded by grants R01AI112920, R21AI147573, R01AI118799, and R01AI121217 from the National Institutes of Health, National Institute of Allergy and Infectious Diseases.

## Conflict of Interest

The authors declare that the research was conducted in the absence of any commercial or financial relationships that could be construed as a potential conflict of interest.

## Publisher’s Note

All claims expressed in this article are solely those of the authors and do not necessarily represent those of their affiliated organizations, or those of the publisher, the editors and the reviewers. Any product that may be evaluated in this article, or claim that may be made by its manufacturer, is not guaranteed or endorsed by the publisher.
