# Supplementary material for: Heterologous production of the adhesin LIC13411 from pathogenic Leptospira facilitates binding of non-pathogenic Leptospira in vitro and in vivo
Source: Front Cell Infect Microbiol. 2022 Jul 22;12:917963. doi: 10.3389/fcimb.2022.917963 (PMC9354625; doi:10.3389/fcimb.2022.917963)
Supplement: Supplementary file 1 [file DataSheet_1.pdf]

Supplementary Material

1.1 Supplementary Tables

Supplementary Table 1. Primers used for cloning, sequencing, and qPCR.

Name	Sequence	Use	Reference
LIC11574_F	GCGGCGCATATGAAGGTTAAAAATAT CCGGAAAGG	cloning LIC11574 in pMaGro	this study
LIC11574_R	GCGGAGTCTAGATTAAGGCTGTTGTT GTTCGTTATCATCC	cloning LIC11574 in pMaGro	this study
LIC13411_F	GCGGAGCATATGGTGACTTTATCGAA TTCCACAAAAAAATATAACGG	cloning LIC13411 in pMaGro	this study
LIC13411_R	GCGGAGTCTAGACTAATCCGCAACGT CATCTTCTAATAAATCC	cloning LIC13411 in pMaGro	this study
pMaOSeqF	GAGTGACACAGGAACACTTAAC	sequencing pMaGro plasmid	(Gaultney *et al.*, 2020)
pMaOSeqR	CCCATTTTCTGGAGTAGGTAGG	sequencing pMaGro plasmid	(Gaultney *et al.*, 2020)
pMaGroSeqF	GCACTCTCACGAATATAGTGC	sequencing pMaGro plasmid	this study
pMaGroSeqR	CCACATATATTCTGTCCACATTTG	sequencing pMaGro plasmid	this study
Lep16s_For	TAGTGAACGGGATTAGATAC	quantification of leptospires	(Bedir *et al.*, 2010)
Lep16s_Rev	GGTCTACTTAATCCGTTAGG	quantification of leptospires	(Bedir *et al.*, 2010)

Supplementary Table 2. Antibodies used for immunoblots.

Antibody	Host	Source	Dilution	Blocking	Wash	2° Antibody	Source	2° Dilution
α-FlaA1	rabbit	D. Haake	1:5,000	5% milk with 3% goat serum in TBS	TBS	gt-α-Rbt-HRP	Promega W401B	1:10,000
α-LipL31	rabbit	D. Haake	1:5,000	5% milk with 3% goat serum in TBS	TBS	gt-α-Rbt-HRP	Promega W401B	1:10,000
α-OmpL47	rabbit	D. Haake	1:5,000	5% milk with 3% goat serum in TBS	TBS	gt-α-Rbt-HRP	Promega W401B	1:10,000
α-LipL21	rabbit	D. Haake	1:5,000	5% milk with 3% goat serum in TBS	TBS	gt-α-Rbt-HRP	Invitrogen A16110	1:25,000
affinity purified: α-LIC11574	mouse	this study	1:250	3% BSA in TBS-T	TBS-T	gt-α-Ms-AP	Promega S372B	1:10,000
affinity purified: α-LIC13411	mouse	this study	1:250	3% BSA in TBS-T	TBS-T	gt-α-Ms-HRP	Promega W402B	1:10,000
α-VE-cadherin	rabbit	Invitrogen #36-1900	1:125	3% BSA in TBS-T	TBS-T	gt-α-Rbt-HRP	Promega W401B	1:5,000
α-phospho-Y658 VE-cadherin	rabbit	Invitrogen #44-1144G	1:500	3% BSA in TBS-T	TBS-T	gt-α-Rbt-HRP	Promega W401B	1:10,000
α-phospho-Y731 VE-cadherin	rabbit	Invitrogen #44-1145G	1:1,000	3% BSA in TBS-T	TBS-T	gt-α-Rbt-HRP	Promega W401B	1:10,000
α-β-actin	mouse	Cell Signaling #3700	1:2,000	3% BSA in TBS-T	TBS-T	gt-α-Ms-HRP	Promega W402B	1:10,000

Supplementary Table 3. Antibodies used for immunofluorescence microscopy.

Antibody	Host	Target	Source (Cat. No.)	Dilution
α-Cadherin 5, Clone 75	mouse	VE-cadherin	BD Bioscience (610252)	1:300
α-MBP	rabbit	MBP	NEB (E8030)	1:10,000
α-*L. biflexa*	rabbit	non-pathogenic *Leptospira*	Biogenesis (5633-4955)	1:20,000
α-*Leptospira* - FITC conjugate	rabbit	pathogenic *Leptospira*	USDA/NVSL (LEP-FAC)	1:100
AlexaFluor 488 α-mouse IgG	goat	mouse IgG	Invitrogen (A11029)	1:1,000
AlexaFluor 568 α-mouse IgG	goat	mouse IgG	Invitrogen (A11031)	1:1,000
AlexaFluor 568 α-rabbit IgG	goat	rabbit IgG	Invitrogen (A11036)	1:1,000

Supplementary Table 4. Raw HMEC binding data.

		**genomes/ 50 µL inoculum**	**genomes bound/well**	**% inoculum bound**			**genomes/ 50 µL inoculum**	**genomes bound/well**	**% inoculum bound**
**Replicate 1**	***Lb* pMaGro**	1.58E+06	8.34E+04	5.52	**Replicate 3**	***Lb* pMaGro**	4.92E+05	1.98E+04	4.63
		1.39E+06	9.18E+04	6.07			4.31E+05	2.21E+04	5.17
		1.76E+06	9.91E+04	6.56			4.64E+05	1.86E+04	4.34
		1.68E+06	N/A	N/A			3.96E+05	N/A	N/A
		1.15E+06	N/A	N/A			3.74E+05	N/A	N/A
		1.52E+06	N/A	N/A			4.14E+05	N/A	N/A
	Lb* pMG. *lic11574	2.32E+06	2.41E+05	10.83		***Lb* pMG. *lic11574***	2.91E+05	2.55E+04	8.09
		2.30E+06	2.30E+05	10.36			3.50E+05	1.62E+04	5.13
		2.05E+06	1.98E+05	8.89			3.06E+05	2.27E+04	7.18
	Lb* pMG. *lic13411	1.07E+06	8.20E+04	8.11		***Lb* pMG. *lic13411***	1.19E+05	1.68E+04	15.18
		1.09E+06	1.01E+05	9.95			9.51E+04	2.05E+04	18.49
		8.70E+05	1.39E+05	13.77			1.18E+05	1.97E+04	17.76
**Replicate 2**	***Lb* pMaGro**	7.33E+05	3.35E+04	3.98	**Replicate 4**	***Lb* pMaGro**	1.09E+06	8.49E+03	1.27
		6.83E+05	1.57E+04	1.86			4.61E+05	1.20E+04	1.80
		7.77E+05	3.09E+04	3.68			8.61E+05	2.18E+04	3.28
		8.88E+05	N/A	N/A			5.08E+05	N/A	N/A
		8.66E+05	N/A	N/A			4.57E+05	N/A	N/A
		1.10E+06	N/A	N/A			6.18E+05	N/A	N/A
	Lb* pMG. *lic11574	1.15E+06	3.62E+04	3.05		***Lb* pMG. *lic11574***	9.31E+05	1.91E+04	1.73
		1.23E+06	3.35E+04	2.83			1.18E+06	1.41E+04	1.28
		1.18E+06	3.70E+04	3.12			1.20E+06	2.09E+04	1.89
	Lb* pMG. *lic13411	4.07E+05	1.84E+04	4.13		***Lb* pMG. *lic13411***	3.56E+05	9.83E+03	2.31
		4.92E+05	1.82E+04	4.10			3.59E+05	2.04E+04	4.80
		4.35E+05	1.94E+04	4.37			5.63E+05	2.61E+04	6.14

N/A = For *Lb* pMaGro, inoculum was quantified in sextuplicate, whereas binding was quantified in triplicate

Supplementary Table 5. Raw VE-cadherin binding data.

		genomes/ 50 µL inoculum	genomes bound/well	% inoculum bound
Replicate 1	*Lb* pMaGro	1.98E+05	1.19E+04	6.16
		1.92E+05	9.38E+03	4.87
		1.88E+05	1.06E+04	5.52
	Lb pMG. *lic11574*	1.33E+05	4.20E+03	3.29
		1.12E+05	4.54E+03	3.55
		1.38E+05	4.45E+03	3.48
	Lb pMG. *lic13411*	8.58E+04	1.07E+04	10.81
		9.97E+04	1.31E+04	13.26
		1.10E+05	1.59E+04	16.14
	Li sv. Manilae	3.78E+04	4.64E+02	1.09
		4.14E+04	4.50E+02	1.06
		4.85E+04	5.22E+02	1.22
Replicate 2	*Lb* pMaGro	2.78E+05	2.48E+04	9.68
		2.30E+05	1.85E+04	7.22
		2.63E+05	2.25E+04	8.74
	Lb pMG. *lic11574*	3.60E+05	2.30E+04	7.77
		2.61E+05	2.63E+04	8.88
		2.68E+05	2.63E+04	8.88
	Lb pMG. *lic13411*	1.04E+05	4.43E+04	39.59
		1.02E+05	4.55E+04	40.70
		1.29E+05	3.44E+04	30.74
	Li sv. Manilae	1.07E+05	4.01E+03	3.81
		1.00E+05	4.19E+03	3.99
		1.08E+05	4.14E+03	3.94
Replicate 3	*Lb* pMaGro	3.88E+05	1.75E+04	5.18
		3.42E+05	1.39E+04	4.12
		2.86E+05	1.76E+04	5.20
	Lb pMG. *lic11574*	2.46E+05	9.76E+03	3.65
		2.67E+05	1.07E+04	4.00
		2.90E+05	1.08E+04	4.04
	Lb pMG. *lic13411*	1.19E+05	1.84E+04	13.59
		1.44E+05	1.97E+04	14.50
		1.44E+05	1.81E+04	13.35
	Li sv. Manilae	1.20E+05	5.84E+03	4.60
		1.28E+05	5.81E+03	4.58
		1.33E+05	6.68E+03	5.27
	Li sv. Copen.	2.95E+05	3.39E+04	12.46
		2.75E+05	3.12E+04	11.47
		2.47E+05	3.55E+04	13.04

		genomes/ 50 µL inoculum	genomes bound/well	% inoculum bound
Replicate 4	*Lb* pMaGro	2.08E+05	5.59E+03	3.06
		1.77E+05	5.01E+03	2.74
		1.62E+05	5.68E+03	3.11
	Lb pMG. *lic11574*	9.07E+04	2.30E+03	3.21
		6.12E+04	1.95E+03	2.72
		6.35E+04	1.65E+03	2.30
	Lb pMG. *lic13411*	2.82E+05	4.66E+04	16.90
		2.97E+05	3.88E+04	14.05
		2.49E+05	2.95E+04	10.69
	Li sv. Manilae	2.36E+05	3.91E+03	1.58
		2.49E+05	4.18E+03	1.69
		2.58E+05	4.34E+03	1.75
	Li sv. Copen.	1.50E+05	2.61E+04	16.42
		1.56E+05	2.22E+04	13.98
		1.70E+05	2.50E+04	15.74
Replicate 5	*Lb* pMaGro	1.49E+05	7.02E+03	3.23
		1.61E+05	5.52E+03	2.54
		3.42E+05	6.39E+03	2.94
	Lb pMG. *lic11574*	5.70E+05	1.04E+04	1.87
		5.95E+05	1.18E+04	2.12
		4.99E+05	1.01E+04	1.82
	Lb pMG. *lic13411*	2.07E+05	1.59E+04	9.21
		1.49E+05	1.67E+04	9.69
		1.62E+05	1.66E+04	9.60
	Li sv. Manilae	1.16E+05	9.99E+02	0.80
		1.26E+05	9.72E+02	0.78
		1.34E+05	1.11E+03	0.89
	Li sv. Copen.	2.11E+05	2.10E+04	10.00
		2.12E+05	1.79E+04	8.51
		2.07E+05	2.39E+04	11.35

1.2 Supplementary Figures

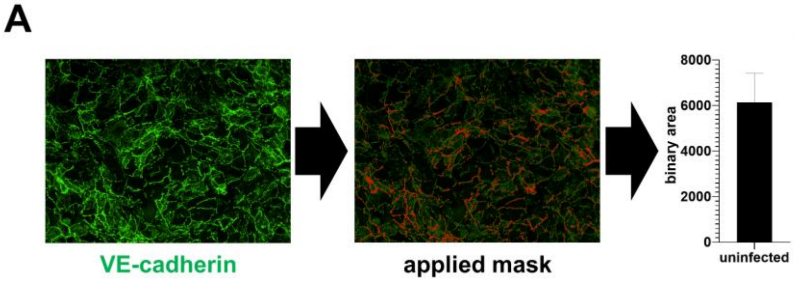

B

Threshold Parameters	
Signal Intensity Min.	2*(mean of control)
Signal Intenisty Max.	4095
Smooth	Off
Clean	1X
Fill Holes	Off
Separate	2X
Size	0 to 26.0
Circularity	Off

Supplementary Figure 1. Quantifying VE-cadherin within AJs. (A) Representative image analyzed by utilizing a binary mask applied to an IF image in Nikon NIS Elements software to quantify VE-cadherin in adherens junctions (AJs). Areas of staining at cell junctions were identified and quantified to determine the binary area covered by these junctional complexes. **(B)** Parameters for creating binary mask were determined after viewing hundreds of images from independent experiments.

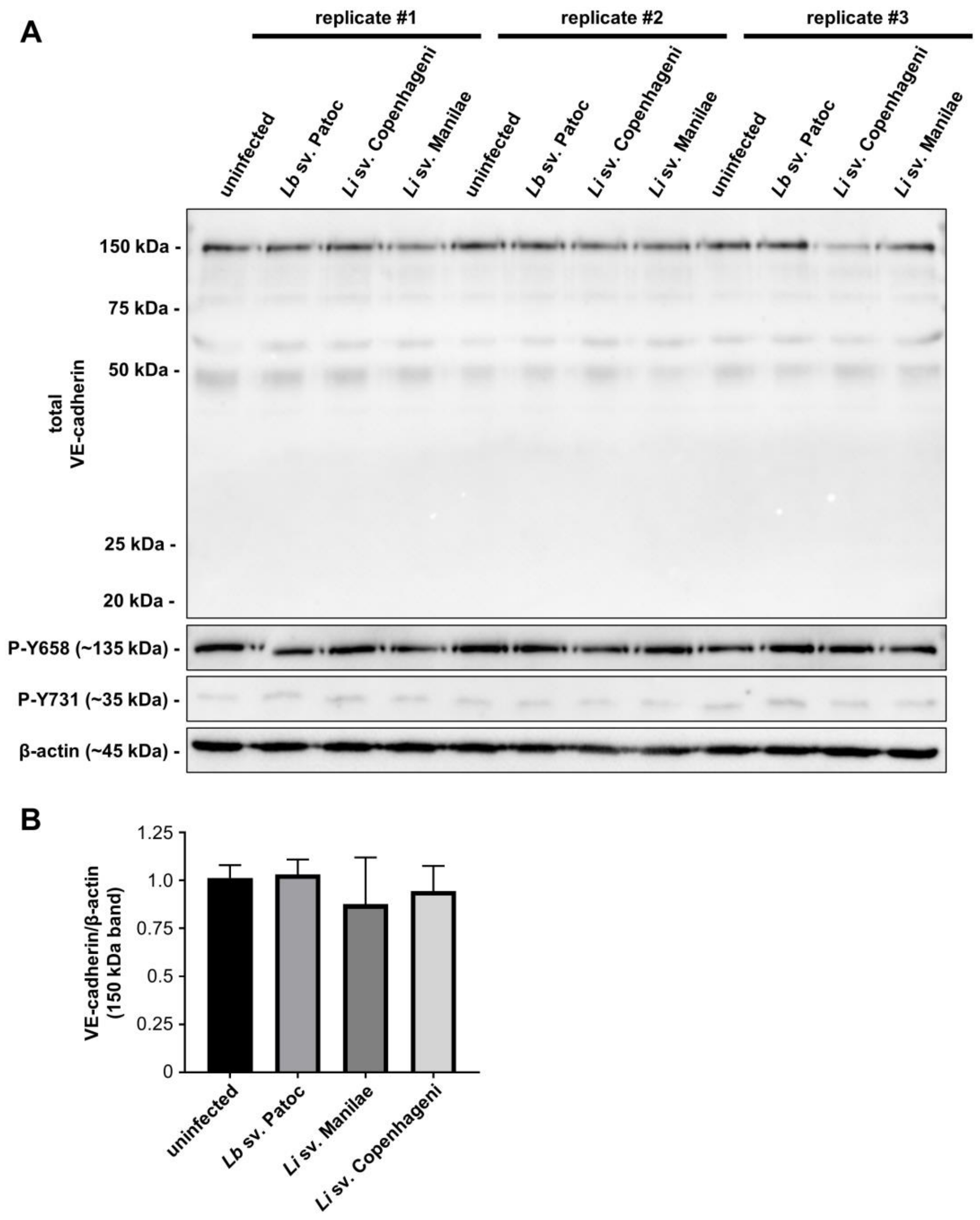

Supplementary Figure 2. *Leptospira* infection of HMECs does not affect levels of VE-cadherin nor the phosphorylation status of VE-cadherin. Cell lysate from uninfected cells, or cells infected with *L. biflexa* sv. Patoc, *L. interrogans* sv. Copenhageni, or *L. interrogans* sv. Manilae were collected. **(A)** Immunoblots were performed on lysate to identify total VE-cadherin, phospho-Y658-VE-cadherin, and phospho-Y731-VE-cadherin. There is no change in total VE-cadherin levels, accumulation of degradation products, or change in phosphorylation at residues Y658 or Y731 upon infection with *Leptospira* spp. An antibody to β-actin was used as a loading control. **(B)** Densitometry was performed on the band corresponding to full length VE-cadherin at 150 kDa and normalized to β-actin. No change in VE-cadherin level was identified upon infection with any strain of *Leptospira*. Mean ± SEM is plotted. Each *Leptospira* infection is compared to the uninfected control.

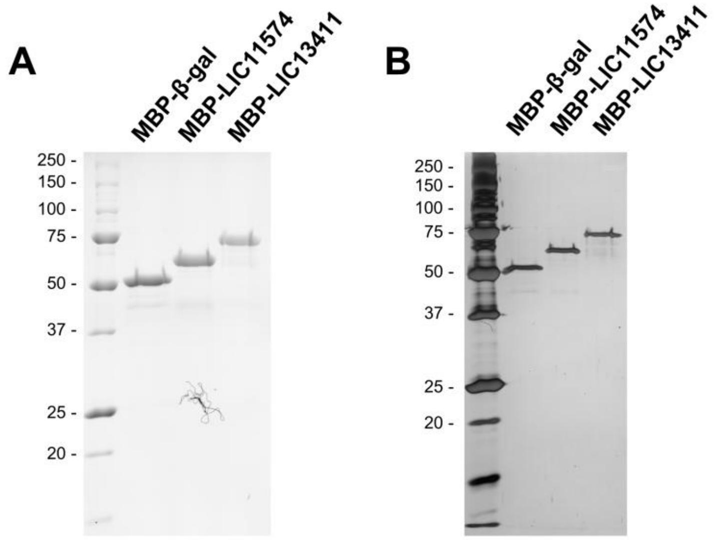

Supplementary Figure 3. Purification of MBP-adhesin fusion proteins. Representative gels demonstrating purity of MBP-adhesin fusions are shown. Expected molecular weights of MBP-β-gal, MBP-LIC11574, and MBP-LIC13411 are 50, 60, and 68 kDa respectively. **(A)** SDS-PAGE was run with 1 µg of protein was loaded per lane and imaged by stain-free methods. **(B)** SDS-PAGE was run with 100 ng of protein was loaded per lane and silver stained.

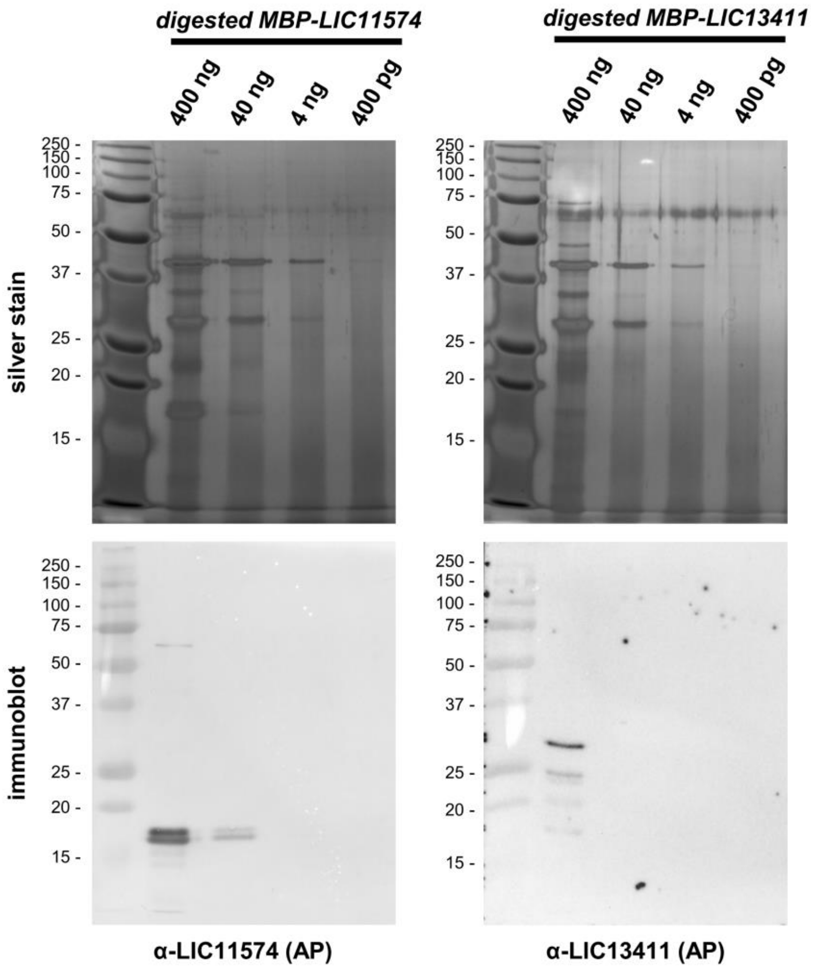

Supplementary Figure 4. Affinity purification of mouse sera against LIC11574 and LIC13411. Sera from mice immunized with MBP-LIC11574 and MBP-LIC13411 was affinity purified. MBP-LIC11574 and MBP-LIC13411 were digested with Factor Xa to remove MBP tag, which has an expected molecular weight of ~43 kDa. Expected molecular weights of LIC11574 and LIC13411 are ~18 kDa and ~26 kDa respectively. Molecular weights of Factor Xa is ~43 kDa, with chains of ~27 and ~16 kDa. Immunoblots of digested MBP-adhesin constructs using affinity purified sera shows reactivity of affinity purified sera against chains corresponding to LIC11574 and LIC13411, with little or no reactivity to MBP tag suggesting specificity for the respective adhesin. Limit of detection using the affinity purified sera is around 40 ng for digested MBP-LIC11574 and 400 ng for digested MBP-LIC13411.

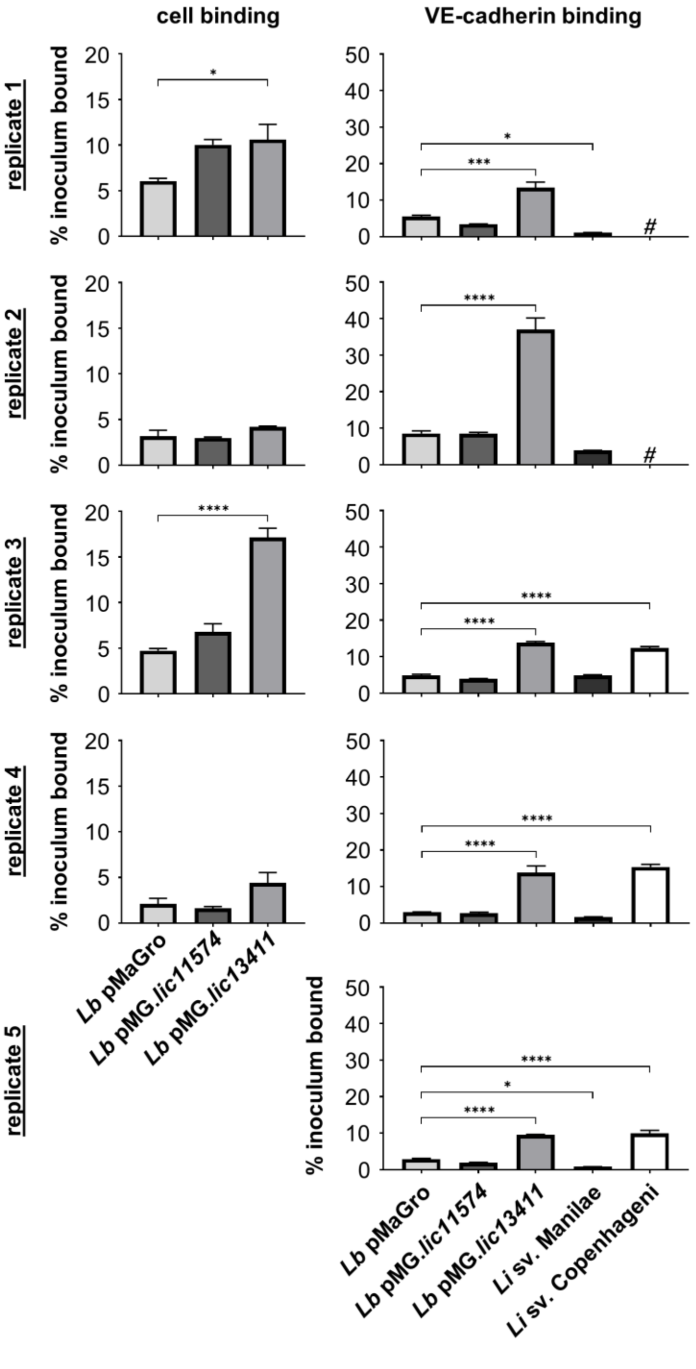

Supplementary Figure 5. Inidvidual replicates of cell and VE-cadherin binding assays. (Left) HMECs were grown to post-confluence, *Leptospira* strains were added at an MOI of 20, incubated for 1 hour, and qPCR was performed to quantify bound bacteria. (Right) VE-cadherin was plated, 3.5 x 10^5 leptospires were added to each well, incubated for 1 hour, and qPCR was performed to quantify bound bacteria. Data from each independent experiment are shown. # denotes that the strain was not used in the experiment. For all data, all technical replicates each day are graphed (n=3). Mean ± SEM is plotted. Each adhesin-encoding strain is compared to the *L. biflexa* pMaGro control. * denotes $p \leq 0.05$, *** denotes $p \leq 0.001$, **** denotes $p \leq 0.0001$.
